# Neuroinvasive Disease and West Nile Virus Infection, North Dakota, USA, 1999–2008

**DOI:** 10.3201/eid1804.111313

**Published:** 2012-04

**Authors:** Paul J. Carson, Stephanie M. Borchardt, Brian Custer, Harry E. Prince, Joan Dunn-Williams, Valerie Winkelman, Leslie Tobler, Brad J. Biggerstaff, Robert Lanciotti, Lyle R. Petersen, Michael P. Busch

**Affiliations:** University of North Dakota School of Medicine and Health Sciences, Grand Forks, North Dakota, USA (P.J. Carson, S.M. Borchardt);; Sanford Health, Fargo, North Dakota, USA (P.J. Carson);; Fargo Veterans Affairs Medical Center, Fargo (S.M. Borchardt);; Wisconsin Department of Health, Madison, Wisconsin, USA (S.M. Borchardt);; Blood Systems Research Institute, San Francisco, California, USA (B. Custer, L. Tobler, M.P. Busch);; Focus Diagnostics, Cypress, California, USA (H.E. Prince);; Creative Testing Solutions, Tempe, Arizona, USA (J. Dunn-Williams, V. Winkelman);; Centers for Disease Control and Prevention, Fort Collins, Colorado, USA (B.J. Biggerstaff, R. Lanciotti, L.R. Petersen);; University of California San Francisco, San Francisco (M.P. Busch)

**Keywords:** West Nile virus, viruses, epidemiology, serology, incidence, blood donors, neuroinvasive disease, meningitis, encephalitis, flaccid paralysis, arbovirus, flavivirus, North Dakota, United States, vector-borne infections

## Abstract

To determine risk for West Nile virus (WNV) neuroinvasive disease in North Dakota, we tested plasma samples from blood donors for WNV IgG and compared infection rates with reported WNV neuroinvasive disease incidence. We estimate that 1 in 244 WNV infections leads to neuroinvasive disease; risk is substantially increased among men and older persons.

Human infection with West Nile virus (WNV) was first identified in North America in 1999 during an outbreak in New York City (NYC), New York, USA (*1*). Since that time, the ArboNET surveillance system housed in the Centers for Disease Control and Prevention (Atlanta, GA, USA) has documented the virus’ spread across the United States (*2*); incidence of disease was highest in the central plains (*3*). Because most persons with WNV infection remain asymptomatic or have West Nile fever, for which routine diagnostic testing is not recommended, the number of infections vastly exceeds the number of cases reported (*4*).

On the basis of an initial study of the 1999 outbreak in NYC, it was estimated that 1 case of neuroinvasive disease (meningitis, encephalitis, or acute flaccid paralysis) occurred for 140 WNV infections (*5*). This ratio is frequently quoted in the literature and has been used to develop national estimates for incidence of WNV infection (*6*). More recent data derived from blood donor screening suggested that WNV neuroinvasive disease (WNND) might occur as frequently as 1 case for 256–353 infections (*7*). Furthermore, surveillance data suggest that the proportion of infected persons in whom WNND develops varies markedly by age and sex (*3*). Previous studies have not clearly identified these differences or used age- and sex-adjusted proportions to estimate the cumulative incidence.

Understanding the full incidence of disease, including asymptomatic infections, is essential for preventing transfusion- and transplantation-associated infections. The purposes of this study were to define the cumulative WNV infection incidence spanning a decade in a highly disease-endemic area and to correlate this with the reported incidence of WNND.

## The Study

Plasma from 6,999 sequential volunteer blood donations collected from November through December 2008 at North Dakota donation centers was archived at Creative Testing Solution’s donor screening laboratory in Tempe, Arizona, USA. Samples were selected to include only those from donors with a North Dakota residence as determined on the basis of postal codes. The resulting 4,514 samples were tested for WNV-specific IgG, and IgM if IgG test results were reactive, by using commercially available ELISAs (*8*) at Focus Laboratories (Cypress, CA, USA).

The cumulative number of infections by age and sex strata in the North Dakota population was estimated by multiplying the corresponding stratum-specific WNV-specific IgG seroprevalence of blood donors by the North Dakota population (US Census Bureau data, 2010). Because blood donors include only persons >16 years of age, the seroprevalence among persons <16 years of age could not be accurately estimated. Therefore, estimates were only calculated for the adult population. For the purposes of this report, adolescents 16–17 years of age are considered adults. The corresponding ratio of WNND cases to infections for each age and sex stratum was determined by dividing the estimated number of infections by the number of WNND cases reported in North Dakota with onset from the first reported case during 2002–2008.

We assessed specificity of the IgG response by examining a subset of 54 samples across the range of IgG responses (every 6th sample) of antibody levels in seropositive donors. We used WNV 90% plaque-reduction neutralization tests and Vero cells as described (*9*).

We calculated relative risks (RRs) and 95% CIs by using standard, asymptotic methods. We performed statistical analyses by using SAS version 9.2 (SAS Institute, Cary, NC, USA), Epi Info (Centers for Disease Control and Prevention), and R version 2.11.1 (www.r-project.org).

Of the 4,514 North Dakota blood donors whose plasma samples were tested, 2,349 (52%) were male. Data on race were available for 4,166 (92%) donors: 98% were white/non-Hispanic, 1% (40) were American Indian, and 0.2% (*10*) were black/non-Hispanic. Among 370 (8.2%) donors who were positive for WNV-specific IgG, 28 (7.5%) were positive for WNV-specific IgM, suggesting recent infection. All 54 representative IgG-reactive samples tested by plaque-reduction neutralization tests had WNV-specific neutralizing antibodies, confirming the specificity of the IgG assay results.

We estimate a total of 44,511 WNV infections in North Dakota residents from its 1999 introduction to the United States through 2008. Seroprevalence was highest among persons 16–24 years of age compared with all other ages combined (RR 1.8, 95% CI 1.2–2.6) and men (9.2%) compared with women (7.2%; RR 1.3, 95% CI 1.1–1.6), possibly reflecting differences in mosquito exposure (Table). Comparison of seroprevalence data with reported numbers of WNND cases in North Dakota indicated that WNND was more likely to develop in infected men than women (RR 1.3, 95% CI 1.0–1.8) and that age was a strong predictor for development of WNND (Table). Among persons ≥65 years of age, the risk was 1 in 54 (95% CI 1:43–1:67), ≈16.0× (95% CI 9.1–28.2) as high as that for persons in the youngest age group. Overall, the risk of WNND development after WNV infection was 1 in 244 (95% CI 1:213–1:286).

**Table:** Correlation of WNV IgG seroprevalence among blood donors with WNND, by sex and age group, North Dakota, USA, 2002–2008*

Sex and age group, y	No. donors	Seroprevalence, % (95% CI)	Seropositivity, RR (95% CI)	Total population†	WNV-infected population‡	No. WNND cases§	Ratio of WNND cases to WNV infections (95% CI)	WNND, RR (95% CI)	Inverse of ratio¶
Male	2,349	9.2 (6.1–8.3)		279,252	26,122	119	0.0045 (0.0038–0.0054)		220
16–24	321	12.8 (9.1–16.4)	Referent	56,156	7,188	10	0.0014 (0.0008–0.0026)	Referent	719
25–44	562	8.7 (6.4–11.1)	0.7 (0.5–1.0)	89,968	7,827	22	0.0028 (0.0018–0.0042)	2.0 (1.0–4.2)	356
45–64	1,132	9.3 (7.6–11.0)	0.7 (0.5–1.0)	90,701	8,435	34	0.0040 (0.0029–0.0056)	2.9 (1.4–5.9)	248
≥65	334	6.3 (3.7–8.9)	0.5 (0.3–0.8)	42.427	2,672	53	0.0198 (0.0152–0.0258)	14.3 (7.3–28.0)	50
Female	2,165	7.2 (6.2–8.3)		271,968	18,353	63	0.0034 (0.0027–0.0044)		291
16–24	316	9.8 (6.5–13.1)	Referent	50,274	4,926	4	0.0008 (0.0003–0.09)	Referent	1,231
25–44	600	6.7 (4.7–8.7)	0.7 (0.4–1.1)	78,869	5,284	16	0.0030 (0.0018–0.0049)	3.7 (1.2–11.1)	330
45–64	1,010	7.5 (5.9–9.2)	0.8 (0.5–1.1)	87,775	6,583	17	0.0026 (0.0016–0.0042)	3.2 (1.1–9.4)	387
≥65	239	2.9 (0.8–5.1)	0.3 (0.1–0.7)	55,050	1,596	26	0.0163 (0.0111–0.0241)	20.1 (7.0–57.4)	61
Total	4,514	8.2 (7.4–9.0)		551,220	44,511	182	0.0041 (0.0035–0.0047)		244

*WNV, West Nile virus; WNND, WNV neuroinvasive disease; RR, relative risk. †Source: US Census Bureau, 2010. ‡Seroprevalence × population. §Data from Centers for Disease Control and Prevention; ArboNET. ¶No. WNV infections/no. WNND cases.

## Conclusions

We estimated that during 1999 through 2008, >40,000 North Dakota residents were infected with WNV. WNND was ≈30% more likely in WNV-infected male than in WNV-infected female donors, and the risk for WNND markedly increased with age. We estimated that the chance of WNND development in WNV-infected persons >65 years of age was ≈1 in 50.

The findings of this large study of WNV expand on findings from previous seroprevalence studies (*5**,**10**–**15*) and suggest that the incidence of WNND cases related to WNV infection in North Dakota is nearly half that estimated during the 1999 NYC epidemic (*5*). These findings are more consistent with those of our previous study (*7*), which estimated 256 infections for every case of WNND nationwide. That study was based on projections of infection rates from the yield of WNV nucleic acid amplification screening in blood donors across the United States, and it correlated those rates with the number of WNND cases nationwide reported to ArboNET in 2003.

The NYC study (*5*) might have overestimated the proportion of infections that resulted in WNND because of a smaller sample size with wider CIs and selection bias resulting from symptomatic persons preferentially enrolling in the survey. Similar to our study, the NYC study indicated that WNND developed in 1 of every 50 infected persons ≥65 years of age (*5*). Published studies have not stratified the risk for WNND by age and sex, which our data indicate are major considerations in projecting overall incidence of WNV infection rates from the number of WNND case reports. The ratio of cases of WNND to cases of WNV infections ranged from 1 in 50 to 1 in 1,231 from the groups with the highest risk (men >65 years of age) to lowest risk (women 16–24 years of age).

Our study has potential limitations. Blood donors might not represent the general population with regard to mosquito exposure. Also, blood donation is limited to persons >16 years of age; therefore, our analysis is limited to the adult population. The completeness of reporting of WNND cases to ArboNET is also unknown and could vary over time. In addition, differences in WNND rates based on race or ethnicity have not been reported in previous studies, and we were unable to explore these differences because the North Dakota blood donor pool was predominantly white.

Results of our study indicate that ≈1 in 12 North Dakota residents has been infected with WNV. The rate of WNV infection was greatest for younger persons and men. Among those infected with WNV, male sex and older age markedly increased the risk for development of WNND; WNND developed in ≈1 of 50 of the infected men >65 years of age. Prevention measures should be particularly targeted to this group during the summer WNV transmission season.
